# Impact of surgical factors on delayed hyponatremia in patients with nonfunctioning pituitary adenoma after endonasal endoscopic transsphenoidal procedure

**DOI:** 10.1007/s12020-022-03164-y

**Published:** 2022-08-19

**Authors:** Haku Tanaka, Fumihiko Nishimura, Kenta Nakase, Miho Kakutani, Shohei Yokoyama, Takayuki Morimoto, Taekyun Kim, Young-Soo Park, Ichiro Nakagawa, Shuichi Yamada, Kentaro Tamura, Ryosuke Matsuda, Yasuhiro Takeshima, Masashi Kotsugi, Hiroyuki Nakase

**Affiliations:** Department of Neurosurgery, Nara Medical University, 840 Shijocho, Kashihara, Nara 634-8522 Japan

**Keywords:** Delayed hyponatremia, Pituitary adenoma, Hypertension, Diabetes insipidus, Meningitis, Endoscopic transsphenoidal surgery

## Abstract

**Purpose:**

Delayed hyponatremia can occur after pituitary surgery, resulting in prolonged hospitalization. However, the influence of surgical factors after such a procedure has not been well established. The impact of surgery and related factors on delayed hyponatremia was investigated.

**Methods:**

This was a retrospective analysis of 137 consecutive patients who underwent transsphenoidal surgery for a nonfunctioning pituitary adenoma between 2008 and 2019. Preoperative (demographics, comorbidities), intraoperative (resection extent, operation time, blood loss volume, cerebrospinal fluid leak, tumor consistency), and postoperative [hematoma, meningitis, diabetes insipidus (DI), hormonal assessment] data were collected, with statistical analysis of each factor performed.

**Results:**

Among the 137 patients, delayed hyponatremia occurred in 31 (22.6%). Multivariate analysis revealed that those with hypertension had a significantly higher likelihood of avoiding delayed hyponatremia (*p* = 0.004). Although no correlations of direct surgical factors with delayed hyponatremia were found, multivariate analysis of indirect surgical factors showed that presence of a firm tumor, transient DI, and meningitis were significantly associated with delayed hyponatremia (*p* = 0.014, 0.001, and 0.047, respectively). There was also a significant association of severe hyponatremia with appearance of symptoms (*p* = 0.002).

**Conclusion:**

There was a tendency for hypertension to be associated with delayed hyponatremia avoidance, with indirect surgical factors including tumor consistency, transient DI, and meningitis found to have an influence on delayed hyponatremia. It was concluded that attention should be given to non-hypertensive patients with a firm tumor, transient DI, or meningitis after pituitary surgery, as delayed hyponatremia may occur.

## Introduction

Delayed hyponatremia is a well-known postoperative complication occurring after transsphenoidal pituitary tumor surgery. It results from temporal derangement of endocrine function, including cortisol deficiency and inappropriate secretion of antidiuretic hormone (ADH), during the postoperative period [1–3]. Development is generally noted on postoperative day (POD) 4–10, with rates of incidence reported to range from 8–35% [3–12], and affected patients can present various clinical manifestations. Previous studies have found that delayed hyponatremia is a common cause of prolonged hospitalization and/or unexpected readmission after hospital discharge in transsphenoidal pituitary tumor surgery cases [3, 13]. Therefore, identification of patients at risk of delayed hyponatremia is important to decrease the incidence of unexpected readmission or prolonged hospital stay.

Commonly reported causes of delayed hyponatremia following an operation include syndrome of inappropriate secretion of antidiuretic hormone (SIADH) and syndrome of inappropriate anti-diuresis (SID), an SIADH like-phenotype with suppressed arginine vasopressin (AVP) (constitutive activation of V2 receptor), cerebral salt-wasting syndrome, exogenous desmopressin administration, hypocortisolemia, and hypothyroidism [10, 14–20]. However, the precise etiology often remains unknown because SIADH and cerebral salt-wasting syndrome are difficult to distinguish [2, 12, 21]. Although the clinical course of affected patients is transient, delayed hyponatremia occurring after transsphenoidal surgery (TSS) requires additional hospital care [13].

In patients who undergo TSS, delayed hyponatremia is not easily recognized and the clinical course is difficult to predict. Most mild cases are asymptomatic, or show non-specific symptoms such as nausea and vomiting, and become symptomatic only if the sodium level drops below 120 mEq/L. [22, 23], while <105 mEq/L is associated with a mortality rate of >50% [24]. Therefore, symptomatic patients require hospitalization so as to prevent neurological complications [7, 25]. Several clinical studies have been conducted to identify reliable predictors of delayed hyponatremia, though no consensus regarding which factors, especially the influence of surgery, are associated with the greatest risk has been reached [7, 10, 26].

The present study was conducted as a retrospective review of our experience with cases of endoscopic endonasal TSS for a nonfunctioning pituitary adenoma (NFPA), with focus on surgical influence and identification of factors having effects on delayed hyponatremia.

## Methods

### Patients

Approval from our institutional research ethics board was obtained for this retrospective study (authorization number 2652). Data from the medical records of 137 consecutive patients who underwent endoscopic endonasal TSS for treatment of an NFPA from November 2008 to January 2019 were analyzed. After receiving informed consent from each, tumor samples were collected and evaluated. All surgeries were performed by a single experienced neurosurgeon who had experience with more than 360 cases of TSS.

### Data collection

Demographic data (age, gender, body mass index), preoperative data (comorbidities, smoking habit, hormonal assessment), preoperative sodium and postoperative nadir sodium concentrations, pituitary tumor characteristics (size, cell type), intraoperative data (extent of resection, operation time, blood loss volume, cerebrospinal fluid leakage, tumor consistency), postoperative data [imaging assessment of hematoma, hyperintensity of pituitary posterior lobe shown by sagittal T1WI, meningitis, diabetes insipidus (DI), hormonal assessment] were retrospectively collected for 137 patients who underwent endoscopic endonasal surgery for removal of an NFPA. Because of the low number of patients with a functioning pituitary adenoma, those were excluded from the present analysis. During the same period, there were 27 patients with functioning pituitary adenomas, of whom only six had delayed hyponatremia. Because of this small number, there was no statistical power and patients with a functioning pituitary adenoma were excluded from the present analysis. The mechanism of delayed hyponatremia may differ between non-functioning and functioning pituitary adenomas, thus only cases with an NFPA were examined to evaluate the impact of surgical factors on delayed hyponatremia. For this study, delayed hyponatremia was defined as a serum sodium concentration ≤135 mEq/L on or after POD 3, while the degree of hyponatremia was evaluated and divided into mild (131–135 mEq/L), moderate (126–130 mEq/L), and severe (≤125 mEq/L) grade.

### Preoperative management

All patients were assessed preoperatively using the protocol of our institution for TSS for an NFPA. Patients with adrenocorticotropic hormone (ACTH) or thyroid-stimulating hormone deficiency underwent adequate replacement with hydrocortisone and levothyroxine prior to the operation. Sex and growth hormones were not replaced before surgery even when a deficiency was identified during the preoperative evaluation.

### Anesthesia and intraoperative management

Anesthesia was induced with a single injection/continuous infusion of propofol and continuous infusion of remifentanil, then maintained by target-controlled infusions of propofol and remifentanil. Orotracheal intubation was facilitated by use of rocuronium, with no additional muscle relaxants administered thereafter. Mean arterial pressure was maintained within a range of 20% of the preoperative value. All patients received 100 mg of hydrocortisone as a steroid supplement and antibiotics intravenously.

Intraoperative volume replacement was performed based on blood loss and urine output at the discretion of the attending anesthesiologist. Surgical influence, such as operation time, extent of resection, blood loss, and intraoperative cerebrospinal fluid leakage, was evaluated. At the end of the operation, all patients were transferred to the intensive care unit after undergoing postoperative computed tomography scans of the brain immediately after surgery before emergence from anesthesia.

### Surgical procedures

All patients underwent endoscopic endonasal transsphenoidal procedures with neuronavigation guidance and visual evoked potential, as well as motor-evoked potential monitoring. After performing a sphenoidotomy individually tailored according to the extent of the tumor, the sella bone was removed. then the dura mater was incised with caution to prevent damage to the pituitary gland. The tumor was removed as much as possible with preservation of the pituitary gland and pituitary stalk. A firm tumor was defined when the tumor could not be removed with curetting and suction, and thus required a sharp dissection [27]. Following removal, Gelfoam^®^ was applied to prevent postoperative hemorrhaging and low-flow cerebrospinal fluid (CSF) leakage. In cases with high-flow CSF leakage and a large sella defect, pedicled vascularized nasoseptal flap reconstruction was required for repair. A sinus balloon was inserted to fix the reconstructed sella floor for several days without lumbar drainage.

### Postoperative management

Sodium chloride solution (0.9%) was given as maintenance fluid postoperatively in all cases, with an infusion rate of 80 mL/h on POD 0 that was reduced to 40 mL/h on POD 1–2, with infusions of fluid usually stopped on POD 3. In patients with pneumocephalus or meningitis, fluid infusion was continued until symptoms improved and finally stopped after confirmation of their disappearance. All patients received 200 mg of hydrocortisone intravenously on POD 0–1 and 100 mg on POD 2, then oral hydrocortisone medication at 50 mg/day on POD 3–4 and 30 mg/day on POD 5–7. When a patient had hypopituitarism or ACTH deficiency, 30 mg/day of hydrocortisone was administered until hypoadrenalism disappeared. In those with permanent hypoadrenalism, hydrocortisone medication at an appropriate dose was continued. For patients who demonstrated DI, desmopressin acetate hydrate was used until its disappearance. Test results for a diagnosis of ACTH deficiency were evaluated at three months after surgery. ACTH deficiency was defined as a morning serum cortisol concentration below 5 μg/dL with a low to normal serum concentration of ACTH. Transient DI was defined as complete symptom resolution in patients shown in subsequent follow-up appointments for up to six months, while those with sustained symptoms requiring use of desmopressin acetate hydrate beyond six months were considered to have permanent DI.

### Statistical analyses

Determination of normality of continuous quantity data with the present sample size was deemed inappropriate. Therefore, data are presented as the median and interquartile range (IQR) (25%, 75%) as a nonparametric representation. Nominal data are presented as frequency (number) and percentage (%). Logistic regression analysis was used to estimate the effects of clinical indicators (patient characteristics, preoperative examination findings, surgical factors, postoperative examination findings) on development of delayed hyponatremia. Statistics were calculated as odds ratio (OR), as well as 95% confidence interval (95% CI) and *P* value. For a multivariable model, since there were 31 cases of delayed hyponatremia, a model was constructed to limit the number of independent variables using the following methods. The first model used univariate analysis to examine significant patient characteristics. In the second model, preoperative examination factors that were significant in univariate analysis results and variables included in the patient characteristics (first model) were added. For the third model, surgical factors shown to be significant in univariate analysis, and variables included in patient characteristics and preoperative examination findings were added. In the fourth model, postoperative examination factors that were significant in univariate analysis results and variables included in patient characteristics, surgical factors, and preoperative examination findings were added. Among the delayed hyponatremia subjects, risk stratification for symptoms in those with hyponatremia determined based on serum level was performed using logistic regression analysis. *P* values <0.05 were considered to indicate statistical significance. All statistical analyses were performed using SPSS for Windows, version 24.0 (IBM Japan, Tokyo, Japan).

## Results

From November 2008 to January 2019, a total of 137 patients [68 females (49.6%), 69 males (50.4%); median age 59.7 years, range 27–91 years] with an NFPA underwent endoscopic endonasal TSS. Delayed hyponatremia occurred in 31 (22.6%) between POD 5 and 10. There were 17 males (54.8%) in the group with delayed hyponatremia and 52 (49.1%) males in the normonatremia group, suggesting no gender dominance. Median body mass index was 22.61 kg/m^2^ in the delayed hyponatremia and 23.5 kg/m^2^ in the normonatremia group, which was not significantly different. Comorbidities were analyzed regardless of whether any factor had an effect on delayed hyponatremia. Multivariate analysis showed a correlation of hypertension with normal natremia (OR = 0.19, *p* = 0.004), but not hyponatremia, suggesting that hypertension has an influence on sustainable normal natremia. No other factors were found to be correlated with hyponatremia. These results are shown in Table 1.

**Table 1:** Patient characteristics

	Group		Univariate	Multivariable
	Delayed hyponatremia	Normonatremia	OR (95% CI), *P*-value	OR (95% CI), *P*-value
Number of cases	31	106		
Demographics				
Age, years	55.0 (46.0–66.0)	64.0 (50.0–71.3)	0.97 (0.95, 1.00), 0.067	−
Males	17 (54.8%)	52 (49.1%)	1.26 (0.57, 2.82), 0.572	−
Body mass index, kg/m^2^	22.6 (20.1–24.7)	23.5 (21.1–25.6)	0.88 (0.74, 1.04), 0.120	−
Recurrence	3 (9.7%)	25 (23.6%)	0.35 (0.10, 1.24), 0.103	−
Smoking habit	8 (25.8%)	19 (17.9%)	1.59 (0.62, 4.10), 0.335	−
Comorbidities				
Diabetes mellitus	1 (3.2%)	9 (8.5%)	0.36 (0.04, 2.95), 0.341	−
Hypertension	4 (12.9%)	46 (43.4%)	0.19 (0.06, 0.59), 0.004	0.19 (0.06, 0.59), 0.004
Dyslipidemia	6 (19.4%)	38 (35.8%)	0.43 (0.16, 1.14), 0.089	−
Cerebrovascular	0 (0.0%)	5 (4.7%)	n.c.	−
Cardiac	1 (3.2%)	5 (4.7%)	0.67 (0.08, 5.99), 0.723	−
Osteoporosis	0 (0.0%)	5 (4.7%)	n.c.	−
Malignancy	2 (6.5%)	7 (6.6%)	0.98 (0.19, 4.95), 0.976	−
Pulmonary	1 (3.2%)	7 (6.6%)	0.47 (0.06, 3.99), 0.490	−
Hepatic	2 (6.5%)	7 (6.6%)	0.98 (0.19, 4.95), 0.976	−
Renal	2 (6.5%)	5 (4.7%)	1.39 (0.26, 7.56), 0.701	−

Descriptive statistics data are presented as median (inter-quartile range), number, and percentage. *OR* odds ratio, *95% CI* 95% confidence interval, *n.c.* not calculated. Logistic regression analysis was performed with group (delayed hyponatremia = 1) as the dependent variable. The multivariable model included factors that were significant in univariate analysis. For continuous variables, OR per one unit increase was calculated

Preoperative blood examination results and tumor size shown by MRI are presented in Table 2. Blood examination findings showed no differences between the hyponatremia and normal natremia groups. Although multivariate analysis showed no significant correlation, univariate analysis revealed a correlation of anterior-posterior distance of the tumor with delayed hyponatremia (OR = 1.07, *p* = 0.025), suggesting a weak association with compression of the pituitary gland posterior lobe.

**Table 2:** Preoperative examination

	Group		Univariate	Multivariate
	Delayed hyponatremia	Normonatremia	OR (95% CI), *P*-value	OR (95% CI), *P*-value
Pre-laboratory tests				
Sodium, mEq/mL	141 (139–142)	141 (139–142)	1.02 (0.90, 1.16), 0.772	−
Potasium, mEq/mL	4.0 (3.9–4.2)	4.1 (3.9–4.3)	0.75 (0.23, 2.46), 0.638	−
Cl, mEq/mL	104 (103–105)	104 (102–105)	1.02 (0.90, 1.15), 0.751	−
BUN	13.0 (10.0–16.0)	15.0 (12.0–17.0)	0.92 (0.84, 1.02), 0.118	−
Cre	0.7 (0.6–0.9)	0.8 (0.6–0.9)	0.87 (0.16, 4.75), 0.875	−
ACTH, pg/mL	24.9 (18.1–40.6)	25.0 (16.0–30.9)	1.02 (1.00, 1.04), 0.139	−
Cortisol, μg/dL	10.0 (7.7–13.3)	10.1 (5.8–13.6)	1.02 (0.94, 1.10), 0.677	
TSH, μU/mL	1.3 (1.0–2.5)	1.8 (0.9–3.0)	0.95 (0.76, 1.19), 0.640	−
FT4, ng/dL	1.0 (0.9–1.2)	1.1 (0.9–1.2)	1.27 (0.26, 6.11), 0.765	−
Preoperative MRI				
Anteroposterior, mm	19.0 (16.5–24.2)	16.5 (14.3–20.4)	1.07 (1.01, 1.14), 0.025	1.06 (1.00, 1.13), 0.059
Transverse, mm	24.0 (20.8–26.7)	21.6 (18.7–25.8)	1.05 (1.00, 1.11), 0.051	−
Craniocaudal, mm	26.4 (22.5–33.1)	24.3 (19.2–31.7)	1.03 (0.98, 1.07), 0.224	−
Tumor volume, cm^3^	6060 (4381–9558)	4464 (2796–7674)	1.03 (0.99, 1.08), 0.107	−

Descriptive statistics data are presented as median (inter-quartile range), number, and percentage. *OR* odds ratio, *95% CI* 95% confidence interval, *n.c.* not calculated. Logistic regression analysis was performed with group (delayed hyponatremia = 1) as the dependent variable. In the multivariable model, factors that were significant in univariate analysis and variables included in patient characteristics (i.e., hypertension) were included in the model. For continuous variables, OR per one unit increase was calculated

Next, factors related to surgery were analyzed. Direct surgical factors, such as operative duration, extent of resection, intraoperative bleeding volume, and CSF leakage, did not show a correlation with delayed hyponatremia. Furthermore, there was no relationship of indirect postoperative surgical factors including sella hematoma and permanent DI noted. However, the presence of a firm tumor (OR = 3.83, *p* = 0.014), transient DI (OR = 6.21, *p* = 0.001), or meningitis (OR = 6.65, *p* = 0.047) was each significantly correlated with occurrence of delayed hyponatremia in multivariate analysis results, suggesting that a greater level of surgical manipulation of the pituitary stalk could lead to transient DI, while meningitis in these cases may be associated with a rebound effect caused by compensatory treatment for dehydration. Postoperative hyperintensity of the pituitary posterior lobe shown by sagittal T1WI was also examined to elucidate the influence of postoperative existence of the posterior lobe on occurrence of delayed hyponatremia, though there was no difference regarding the percentage of hypersignals between the groups (*p* = 0.097). These results are presented in Table 3.

**Table 3:** Surgical factors

	Group		Univariate	Multivariate
	Delayed hyponatremia	Normonatremia	OR (95% CI), *P*-value	OR (95% CI), *P*-value
Direct surgical factors				
Operation time, min	175 (159–254)	182 (146–222)	1.00 (1.00, 1.01), 0.346	−
Bleeding volume, mL	10.0 (10.0–75.0)	10.0 (10.0–76.3)	1.00 (1.00, 1.00), 0.452	−
Gross total removal	22 (71.0%)	69 (65.1%)	1.31 (0.55, 3.14), 0.543	−
CSF leakage	14 (45.2%)	36 (34.0%)	1.60 (0.71, 3.61), 0.257	−
Indirect surgical factors				
Firm tumor	15 (48.4%)	17 (16.0%)	4.91 (2.05, 11.77), <0.001	3.83 (1.31, 11.20) 0.014
Transient DI	11 (35.5%)	9 (8.5%)	5.93 (2.17, 16.18), 0.001	6.21 (2.05, 18.76), 0.001
Permanent DI	1 (3.2%)	3 (2.8%)	1.14 (0.12, 11.41), 0.908	−
Postoperative bleeding	11 (35.5%)	17 (16.0%)	2.79 (0.92, 8.43), 0.070	−
Meningitis	7 (22.6%)	2 (1.9%)	9.90 (1.04, 94.36), 0.046	6.65 (1.03, 43.03), 0.047
Pituitary hypersignal in T1WI sagittal section	23 (74.2%)	93 (87.7%)	0.33 (0.09, 1.23), 0.097	−

Descriptive statistics data are presented as median (inter-quartile range), number and percentage. *OR* odds ratio, *95% CI* 95% confidence interval, *n.c.* not calculated. Logistic regression analysis was performed with group (delayed hyponatremia = 1) as the dependent variable. In the multivariable model, factors that were significant in univariate analysis, and variables included in patient characteristics and preoperative examination factors (i.e., hypertension, anterior-posterior distance) were included in the model. For continuous variables, OR per one unit increase was calculated

Postoperative blood examination results are shown in Table 4. Univariate analysis revealed a significantly higher value for potassium in the hyponatremia as compared to the normal natremia group (OR = 6.31, *p* = 0.030), while chloride was significantly lower in the hyponatremia group (OR = 0.49, *p* = 0.001). Multivariate analysis as well revealed that chloride had a significantly lower value in the patients with hyponatremia (OR = 0.36, *p* < 0.001).

**Table 4:** Postoperative examination

	Group		Univariate	Multivariate
	Delayed hyponatremia	Normonatremia	OR (95% CI), *P*-value	OR (95% CI), *P*-value
Postoperative tests				
Sodium, mEq/mL	128 (119–133)	142 (140–144)	-	
Potassium, mEq/mL	4.1 (3.9–4.2)	3.9 (3.6–4.1)	6.31 (1.20, 33.28), 0.030	0.51 (0.02, 12.46), 0.679
Cl, mEq/mL	91 (85–97)	104 (102–106)	0.49 (0.32, 0.74), 0.001	0.36 (0.21, 0.63), <0.001
BUN	12.0 (9.0–15.0)	14.0 (11.0–17.0)	0.92 (0.82, 1.03), 0.158	
Cre	0.6 (0.5–0.8)	0.7 (0.6–0.9)	0.08 (0.01, 1.08), 0.057	
ACTH, pg/mL	13.1 (5.9–22.5)	23.0 (11.4–32.7)	0.98 (0.95, 1.02), 0.308	
Cortisol, μg/dL	7.4 (2.7–13.0)	8.7 (4.4–13.1)	0.94 (0.84, 1.06), 0.324	
TSH, μU/mL	1.5 (0.3–2.5)	1.2 (0.4–2.0)	0.91 (0.70, 1.18), 0.468	
FT4, ng/dL	1.2 (0.9–1.3)	1.1 (1.0–1.3)	0.52 (0.09, 3.07), 0.474	

Descriptive statistics data are presented as median (inter-quartile range), number, and percentage. *OR* odds ratio, *95% CI* 95% confidence interval, *n.c.* not calculated. Logistic regression analysis was performed with group (delayed hyponatremia = 1) as the dependent variable. In the multivariable model, factors that were significant in univariate analysis and variables included in patient characteristics, surgical factors, and preoperative examination factors (i.e., hypertension, anterior-posterior distance, firm tumor, transient DI, and meningitis) were included in the model. For continuous variables, OR per one unit increase was calculated

Finally, whether the degree of hyponatremia was correlated with symptoms, such as headache, vomiting, and/or nausea, was examined. Among the patients with delayed hyponatremia, 10 were classified as mild (131–135 mEq/L), 7 as moderate (126–130 mEq/L), and 14 as severe (≤125 mEq/L) (Table 5). Symptomatic hyponatremia was noted in 2 (20%) with mild hyponatremia, 3 (42.9%) with moderate, and 13 (92.9%) with severe hyponatremia, indicating a statistically significant occurrence of symptomatic hyponatremia in the severe group (OR = 52, *p* = 0.002). These results are presented in Table 5.

**Table 5:** Risk stratification for symptoms in patients with hyponatremia based on serum sodium level

	No.	Symptomatic (%)	OR (95% CI)	*P*-value
Mild (131–135 mEq/L)	10	2 (20.0)	1.00 (reference)	
Moderate (126–130 mEq/L)	7	3 (42.9)	3.00 (0.35, 25.87)	0.318
Severe (≤125 mEq/L)	14	13 (92.9)	52.00 (4.03, 670.60)	0.002

*OR* odds ratio, *95% CI* 95% confidence interval. Logistic regression analysis was performed with group (symptomatic delayed hyponatremia = 1) as the dependent variable

## Discussion

Postoperative complications following TSS are generally associated with sodium disturbance, such as DI and delayed hyponatremia [3–12, 28]. A triphasic pattern of polyuria, oliguria, and polyuria after surgical procedures for tumors in the region of the hypophysis and hypothalamus has been confirmed in previous studies [29, 30]. Oliguria frequently leads to hyponatremia, since water intake is not appropriately reduced [5]. Nevertheless, the precise mechanism of delayed hyponatremia and impact of surgical factors has not been established.

The present study was conducted to determine factors related to delayed hyponatremia following endonasal endoscopic TSS in patients with an NFPA. Of 137 patients who underwent that surgical procedure, 31 (22.6%) developed delayed hyponatremia, with equal frequency in females and males, though female gender has been reported to be a risk factor [10]. There were no differences between the delayed hyponatremia and normal natremia groups in the present study in regard to age, BMI, smoking habit, or rate of recurrence following a previous pituitary surgical procedure.

The associations of comorbidities with occurrence of delayed hyponatremia were also examined. Although a previous report stated that patients with a preexisting renal disorder had a higher likelihood of developing delayed hyponatremia as compared to those without [31], multivariate analysis conducted in the present study revealed that a preexisting renal disorder did not have a significant association, though interestingly, patients with preexisting hypertension had a statistically higher likelihood of avoiding delayed hyponatremia. The mechanism of this association with hypertension and delayed hyponatremia remains unclear, and requires further investigation.

Although there was no significant correlation shown by multivariate analysis, univariate analysis results revealed that the anterior-posterior distance of the tumor was correlated with delayed hyponatremia (*p* = 0.025), suggesting a weak association with compression of the pituitary gland posterior lobe, which might cause inappropriate release of AVP following surgery. Previous studies have also noted that patients with a large tumor frequently developed delayed hyponatremia as compared to those without [3, 4, 7, 10, 11] and speculated that a larger size tumor was highly likely to compress or damage the pituitary stalk above the pituitary gland, and could interfere with AVP secretion [29]. It has also been noted that pituitary function recovery in the delayed phase after surgery might cause a surge of AVP release and fluid retention [4].

In the present study, the influence of surgical factors on occurrence of delayed hyponatremia in the present cohort received focus. Multivariate analysis showed that direct surgical factors, such as operative duration, extent of resection, intraoperative bleeding volume, and intraoperative CSF leakage, had no association with its occurrence. In contrast, the presence of a firm tumor, transient DI, or meningitis, as well as indirect surgical factors each had a significant relationship with occurrence of delayed hyponatremia. These results suggested that a greater level of surgical manipulation of the pituitary stalk could result in dysregulation of AVP secretion and be associated with a higher likelihood of postoperative DI [30]. Meningitis causing a high fever leads to dehydration, which can trigger secretion of AVP, thus affected patients were usually treated with hydration and antibiotics. As a result of hydration treatment, there might have been an association of meningitis with delayed hyponatremia.

Tumor consistency is an important factor for pituitary surgery, as a firm tumor cannot be removed with curetting and suction, and a sharp dissection is necessary, which might cause damage to the pituitary stalk. Such damage to the pituitary stalk has been reported to potentiate post-surgical disturbance of water and electrolytes [32]. Although the present study found no statistical association between gross total removal and delayed hyponatremia, the presence of a firm tumor and transient DI each had a significant relationship with delayed hyponatremia occurrence, suggesting that these two factors might be associated with pituitary stalk damage.

Overall, transient DI appears to be the most common complication after TSS in these cases, with symptomatic hyponatremia the second most common [17, 33]. A previous retrospective study found delayed hyponatremia to be the most common cause for readmission, followed by DI [13]. Other reports have noted occurrence of early transient DI after TSS ranging from 10% to 60% [2, 34, 35]. In the present study, transient DI occurred in 35.5% of the delayed hyponatremia cases and in 8.5% of cases with normal natremia, while multivariate analysis showed that transient DI was significantly correlated with delayed hyponatremia (OR 6.21, *p* = 0.001) as was meningitis (OR 12.03, *p* = 0.006). These results were compatible with previous reports noting that hyponatremia following DI was caused by SIADH due to unregulated release of AVP from denervated posterior pituitary nerve terminals [36, 37].

Postoperative hyperintensity of the pituitary posterior lobe shown by sagittal T1WI was also evaluated to examine the influence of existence of the posterior lobe following surgery on occurrence of delayed hyponatremia. There was no significant difference between the groups regarding hypersignal percentage (*p* = 0.097). Data obtained with AVP sampling are not shown, as they were not useful for determining a diagnosis of DI. Furthermore, postoperative measurements of AVP (data not shown) also indicated no significant differences for AVP values (*p* = 0.440) and no excess production of AVP, such as SIADH. Thus, prediction of individual patient risk for DI or SIADH remains difficult [38].

As for postoperative blood examination evaluations, multivariate analysis revealed that chloride was significantly lower in the hyponatremia as compared to the normal natremia group (*p* < 0.001), suggesting that chloride concentration in the renal tubules is tightly coupled with sodium and water transport [36]. Misono proposed that chloride-mediated feedback could play a role in atrial natriuretic peptide (ANP)-induced natriuresis in patients with a high level of circulating ANP [39].

We also evaluated whether the degree of hyponatremia was correlated to appearance of symptoms, such as headache, vomiting, and /or nausea, and the results showed that patients with severe hyponatremia presented such symptoms with significant frequency (OR = 52, *p* = 0.002). These patients should be treated as soon as possible to avoid deterioration and several reports regarding treatment of delayed hyponatremia to prevent severe symptoms have been presented [25, 40–42], with the latter by Deaver et al. noting that mild fluid restriction (up to 1.5 liters daily) was an effective approach for preventing readmission for hyponatremia after TSS for a pituitary adenoma. Based on the report by D. J. Cote [43], restrictions of fluid (to 1.0 liters daily) and oral salt intake (3–6 g/day) were used for patients in the present cohort with delayed hyponatremia to improve symptoms. Finally, though treatment with the oral vasopressin receptor antagonist tolvaptan can be useful for cases with SIADH [9], that drug was not available in Japan until 2020.

This study has some limitations, including inherent bias related to a retrospective review of cases. Also, this was a single-center study, potentially introducing bias to the results. All of the present patients underwent TSS with an endoscopic approach, thus, though direct surgical factors did not have an influence on delayed hyponatremia, factors with effects identified in this study were not free from confounding effects caused by differences in surgical techniques. All surgeries analyzed in this study were performed by a single neurosurgeon. Because clinical outcomes after TSS can be easily influenced by factors related to the performing physician [44], differences in operator experience and techniques may have had effects on the present results.

## Conclusion

Hypertension showed a tendency to influence avoidance of delayed hyponatremia after pituitary surgery in the present cases. While direct surgical factors were not found to have an association with delayed hyponatremia, indirect surgical factors that included tumor consistency, transient DI, and meningitis did demonstrate such a relationship. Therefore, attention should be given to non-hypertensive patients with a firm tumor, transient DI, or meningitis following pituitary surgery for possible occurrence of delayed hyponatremia.
